# Quantitative multiplex real-time polymerase chain reaction assay for the detection of *Helicobacter pylori* and clarithromycin resistance

**DOI:** 10.1186/s12866-023-02868-z

**Published:** 2023-05-27

**Authors:** Ilsoo Kim, Lee-So Maeng, Joon Sung Kim, Byung-Wook Kim, Dae Young Cheung, Jin Il Kim, Soo-heon Park

**Affiliations:** 1 Divison of Gastroenterology, Department of Internal Medicine, Incheon St. Mary’s Hospital, College of Medicine, The Catholic University of Korea, Incheon, Republic of Korea; 2 Department of Hospital Pathology, Incheon St. Mary’s Hospital, College of Medicine, The Catholic University of Korea, Seoul, Korea; 3 Division of Gastroenterology, Department of Internal Medicine, Yeouido St. Mary’s Hospital, College of Medicine, The Catholic University of Korea, Seoul, Republic of Korea

**Keywords:** *Helicobacter Pylori*, Molecular diagnosis, Macrolide Resistance

## Abstract

**Background:**

Identifying clarithromycin resistance is essential for eradicating *Helicobacter pylori* (HP). Therefore, we evaluated the performance of Allplex™ *H.pylori* & ClariR Assay (Allplex™) for diagnosing and detecting clarithromycin resistance in HP.

**Methods:**

Subjects who underwent esophagogastroduodenoscopy between April 2020 and August 2021 at Incheon St. Mary’s hospital were enrolled in this study. The diagnostic performances of Allplex™ and dual priming oligonucleotide (DPO)-based multiplex polymerase chain reaction (PCR) were compared with sequencing as the gold standard.

**Results:**

A total of 142 gastric biopsy samples were analyzed. Gene sequencing revealed 124 HP infections, 42 A2143G mutations, 2 A2142G mutations, one dual mutation, and no A2142C mutation. DPO-PCR showed 96.0% sensitivity and 100.0% specificity for HP detection; the corresponding rates for Allplex™ were 99.2% and 100.0%. DPO-PCR showed 88.3% sensitivity and 82.0% specificity for A2143G mutation, and Allplex™ showed 97.6% and 96.0%. The Cohen’s Kappa coefficient for overall test results was 0.56 for DPO-PCR and 0.95 for Allplex™.

**Conclusion:**

Allplex™ showed comparable diagnostic performance with direct gene sequencing and non-inferior diagnostic performance to DPO-PCR. Further research is required to confirm whether Allplex™ is an effective diagnostic tool for the eradication of HP.

## Introduction

*Helicobacter pylori* (HP) is one of the most common bacterial infections globally, with a prevalence of approximately 50%. [1] HP is the primary cause of gastric cancer, peptic ulcer disease, and non-ulcer dyspepsia, and its eradication has proven to diminish the incidence of gastric cancer [2] International guidelines recommend eradicating HP when its presence is confirmed [3–5] A triple therapy regimen consisting of clarithromycin was traditionally used for eradication. However, the efficacy of triple therapy has declined recently due to increasing resistance to clarithromycin [6] Consequently, numerous guidelines recommend avoiding the triple therapy regimen in regions where clarithromycin resistance exceeds 15% [5, 7].

Point mutation of HP’s 23 S ribosomal RNA (rRNA) gene limits the access of clarithromycin to the ribosome targets, which is the leading cause of resistance. The most commonly cited point mutations in HP with clarithromycin resistance are two nucleotides in the 23 S rRNA; an adenine-to-guanine transition at either 2142 (A2142G) or 2143 (A2143G) or an adenosine-cytosine transversion at 2142 (A2142C)[8]. These sites account for more than 80% of clarithromycin resistance in HP infection [9].

Dual priming oligonucleotide (DPO)-based multiplex polymerase chain reaction (PCR) (Seeplex *H. pylori*-ClaR ACE Detection; Seegene Inc., Seoul, Korea) was developed for diagnosing HP infection and detects the two point mutations for clarithromycin resistance (A2142G and A2143G) [10]. Tailored therapy based on DPO-PCR results demonstrated higher eradication rates with improved cost-effectiveness compared with standard triple therapy [11].

Allplex *H pylori* and ClariR Assay (Allplex™, Seegene Inc., Seoul, Korea) is a quantitative multiplex PCR for detecting HP and its point mutations on clarithromycin resistance. Allplex™ can detect A2142G, A2143G, and A2142C mutations. A recent study of 180 gastric biopsies reported 100% sensitivity, 97.6% specificity, 98% positive predictive value (PPV), and 100% negative predictive value (NPV) for Allplex™ compared with gene sequencing [12]. To the best of our knowledge, no study compared the diagnostic performance of Allplex™ and DPO-PCR. Therefore, the objective of this study was to investigate the diagnostic performance of Allplex™ and DPO-PCR and compare their performance with that of direct gene sequencing for HP diagnosis and detection of clarithromycin resistance in HP.

## Materials and methods

### Patients

A total of 142 patients who underwent biopsy during upper gastrointestinal endoscopy for diagnosis of HP infection between April 2020 and August 2021 at Incheon St. Mary’s hospital were enrolled in this study. The gastric biopsy specimens included at least one piece from the antrum and body of the stomach. The biopsy specimens were subjected to DPO-PCR and Allplex™ and direct gene sequencing.

All the participants provided informed consent, and this study was approved by the Institutional Review Board (IRB) of Incheon St. Mary’s Hospital, Incheon, South Korea. (OC21DISI0114)

### HP DNA extraction

DNA was isolated by the QIAamp DNA mini kit (Qiagen, Germany) from the gastric biopsy tissue. The tissues were placed in a microcentrifuge tube, and buffer ATL (180 µ), and proteinase K (20 µl) were added. The tube was vortexed and incubated at 56 °C until the tissues were completely lysed. Buffer AL (200 µl) was added to the tube, which was subsequently incubated at 70 °C for 10 min. Next, 240 µl of 100% ethanol was added to the tubes and mixed by vortexing for 15 s. Each tube was placed in a QIAamp spin column and centrifuged at 8,000 rpm for one minute. The columns were washed with AW1 buffer (500 µl) and centrifuged at 8,000 rpm for one minute. AW2 buffer (500 µl) was added to the column and centrifuged at 14,000 rpm for three minutes. Buffer AE (200 µl) was added to each sample and incubated before centrifugation at 8,000 rpm for one minute. Finally, the DNA was extracted from the tissue.

**Fig. 1:**
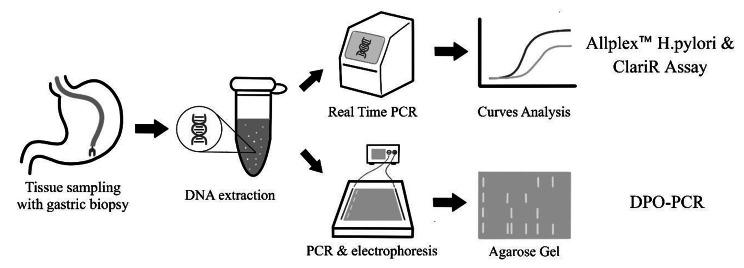
Diagrammatic presentation of the study concept. Abbreviations: DPO-PCR, dual priming oligonucleotide-based multiplex polymerase chain reaction; PCR, Polymerase Chain Reaction; DNA, Deoxyribonucleic acid

To analyze the nucleotide conservation in the region of 23S rRNA positions of 2142 and 2143, we amplified a segment of 546 bp using PCR primers (5’ – GTCCCTCCCGACTGTTTACC – 3, 5’-AACCGCAATGAGCCAACC-3’). The Allplex™ and DPO-PCR were used to detect *H. pylori* and its mutations according to the manufacturer’s recommendations [13]. These tests were performed as previously published [10, 12] (Fig. 1).

### Outcomes

The primary outcome was the diagnostic performance of Allplex™ and DPO-PCR for detecting HP infection. The secondary outcome was the diagnostic performance of Allplex™ and DPO-PCR in detecting the point mutations A2142G and A2143G. Both outcomes were assessed using direct gene sequencing results as reference standards.

### Statistical analysis

The sensitivity, specificity, positive predictive value (PPV), and negative predictive value (NPV) of each test were investigated. Continuous variables were described as means (± SD) or medians (interquartile range) and compared using Student’s t-test. Categorical variables were expressed as numbers (percentages) and analyzed using the chi-square and trend tests. Agreement between the different PCR methods from each test was estimated with the kappa coefficient. All statistical analyses were performed with SPSS software, version 24.0 (SPSS Inc., Chicago, Ill).

## Results

### Patient characteristics

The patients were between 21 and 83 years of age with a median age of 62. Approximately half of the subjects were male (n = 75, 51.8%). The most common indication for biopsy was atrophic gastritis. Intestinal metaplasia was suspected in 119 patients (83.8%). The most common indication for endoscopy was screening (43.0%). (Table 1.)

**Table 1:** Baseline characteristics of the study population

Characteristics	Total (n = 142)
Age (years)	61.3 ± 11.0
Male	75 (51.8)
Comorbidity	
Hypertension	56 (39.4)
Diabetes Mellitus	33 (23.2)
Dyslipidemia	32 (22.5)
BMI	20.3 ± 10.5
Smoking	34 (23.9)
Alcohol	44 (31.0)
EGD indication	
Symptom ^†^	56 (39.4)
Screening	61 (43.0)
Alarm feature ^‡^	18 (12.7)
Surveillance	3 (2.1)
Endoscopic biopsy indication	
Atrophic gastritis	141 (99.3)
Intestinal metaplasia	119 (83.8)
Peptic ulcer	11 (7.7)
Neoplasm of stomach	55 (38.7)

† Symptoms include dyspepsia, reflux symptom, and abdominal pain

‡ Alarm features include anemia, dysphagia, weight loss, GI bleeding, vomiting and bowel habit change

Abbreviations: BMI; Body mass index, EGD; Esophagogastroduodenoscopy

Data are presented as number of patients (%) or mean ± SD.

### Diagnostic performance for HP

The gene sequencing revealed 125 HP infections among the142 subjects, DPO-PCR and Allplex™ found 120 and 124, respectively (Table 2). DPO-PCR showed 96.0% sensitivity, 100.0% specificity, 100.0% PPV, 77.3% NPV, 96.5% accuracy, and 0.85 Cohen’s Kappa coefficient. Allplex™ showed 99.2% sensitivity, 100.0% specificity, 100.0% PPV, 94.4% NPV, 99.3% accuracy, and 0.97 Cohen’s Kappa coefficient. Allplex™ showed higher sensitivity, NPV, and accuracy for HP diagnosis compared with DPO-PCR.

**Table 2:** Performance of the Allplex™*H pylori* and ClariR Assay and DPO-PCR in *H. pylori* detection compared with gene sequencing

	No. (%) of results by reference standard					Analytical performance (%)^‡^					
	TP	TN	FP	FN		Sensitivity	Specificity	PPV	NPV	Accuracy	Kappa (κ)^†^
Allplex™	124 (98.3)	17 (12.0)	0 (0)	1 (0.7)		99.2 (95.6–100.0)	100 (80.5–100)	100	94.4 (70.7–99.2)	99.3 (96.1–100)	0.97 (0.91-1)
DPO-PCR	120 (84.5)	17 (12.0)	0 (0)	5 (3.5)		96.0 (90.9–98.7)	100 (80.5–100)	100	77.3 (59.0-88.9)	96.5 (92.0-98.9)	0.85 (0.72–0.98)

† Cohen’s kappa coefficient, Kappa values representing levels of agreement are categorized as follows: >0.90, almost perfect; 0.80 to 0.90, strong, 0.60 to 0.79, moderate; 0.40 to 0.59, week; 0.21 to 0.39, minimal; (0 to 0.20), none

‡ Values in parentheses are 95% confidence intervals

Abbreviations: Allplex™, Allplex™*H pylori* and ClariR Assay; DPO-PCR, dual priming oligonucleotide-based multiplex polymerase chain reaction; TP, true positive; TN, true negative; FP, false positive; FN, false negative; PPV, positive predictive value; NPV, negative predictive value

### Diagnostic performance for clarithromycin resistance

Of the 142 subjects, gene sequencing discriminated 42 mutations of A2143G and two mutations of A2142G, but there was no A2142C mutation (Table 3). One strain showed both A2143G and A2142G mutations simultaneously. DPO-PCR showed 88.3% sensitivity, 82.0% specificity, 66.0% PPV, 92.1% NPV, 8.4% accuracy, and 0.61 Cohen’s Kappa coefficient for detection of A2143G mutation. Allplex™ showed 97.6% sensitivity, 96.0% specificity, 91.1% PPV, 99.0% NPV, 96.5% accuracy, and 0.92 Cohen’s Kappa coefficient. DPO-PCR showed 50.0% sensitivity, 97.9% specificity, 25.0% PPV, 99.3% NPV, 97.2% accuracy, and 0.62 of Cohen’s Kappa coefficient for detection of A2142G mutation. The corresponding rates for Allplex™ were all 100.0% and 1.00 of Cohen’s Kappa coefficient. The Allplex™ showed superior accuracy compared to that of DPO-PCR for detecting A2142G mutation. (Table 4) The Cohen’s Kappa coefficient was estimated at 0.56 for DPO-PCR and 0.95 for Allplex™ in the overall test results.

**Table 3:** Performance of Allplex™*H pylori* and ClariR Assay and DPO-PCR for detection of A2143G mutation compared with gene sequencing

Tests performances	Allplex™	DPO-PCR
Total positive results of the reference method (n)	42	42
Total positive results of the used test (n)	45	47
Total negative results of the used test (n)	97	95
False positive (n)	4	18
False negative (n)	1	7
Sensitivity %, (95% CI)	97.6 (87.4–99.9)	88.3 (68.6–93.0)
Specificity %, (95% CI)	96.0 (90.1–98.9)	82.0 (73.1–89.0)
Positive predictive value %, (95%CI)	91.1 (79.7–96.4)	66.0 (55.6–75.1)
Negative predictive value %, (95% CI)	99.0 (93.3–99.9)	92.1 (85.6–95.9)
Test Accuracy %, (95% CI)	96.5 (92.0-98.9)	82.4 (75.1–88.3)
Kappa (κ) (95% CI)^†^	0.92 (0.85–0.99)	0.61 (0.47–0.75)

† Cohen’s kappa coefficient, Kappa values representing levels of agreement are categorized as follows: >0.90, almost perfect; 0.80 to 0.90, strong, 0.60 to 0.79, moderate; 0.40 to 0.59, week; 0.21 to 0.39, minimal; (0 to 0.20), none

Abbreviations: Allplex™, Allplex™*H pylori* and ClariR Assay; DPO-PCR, dual priming oligonucleotide-based multiplex polymerase chain reaction; CI, confidence interval

**Table 4:** Performance of Allplex™*H pylori* and ClariR Assay and DPO-PCR for detection of A2142G mutation compared with gene sequencing

Tests performances	Allplex™	DPO-PCR
Total positive results of the reference method (n)	2	2
Total positive results of the used test (n)	2	4
Total negative results of the used test (n)	140	138
False positive (n)	0	3
False negative (n)	0	1
Sensitivity %, (95% CI)	100 (15.8–100)	50.0 (1.3–98.7)
Specificity %, (95% CI)	100 (97.4–100)	97.9 (93.9–99.6)
Positive predictive value %, (95%CI)	100	25.0 (5.3–66.4)
Negative predictive value %, (95% CI)	100	99.3 (97.2–99.8)
Test Accuracy %, (95% CI)	100 (97.4–100)	97.2 (92.9–99.2)
Kappa (κ) (95% CI)^†^	1.00 (1.00–1.00)	0.62 (0.00-0.98)

† Cohen’s kappa coefficient, Kappa values representing levels of agreement are categorized as follows: >0.90, almost perfect; 0.80 to 0.90, strong, 0.60 to 0.79, moderate; 0.40 to 0.59, week; 0.21 to 0.39, minimal; (0 to 0.20), none

Abbreviations: Allplex™, Allplex™*H pylori* and ClariR Assay; DPO-PCR, dual priming oligonucleotide-based multiplex polymerase chain reaction; CI, confidence interval

### Discordance between the PCR methods

**Fig. 2:**
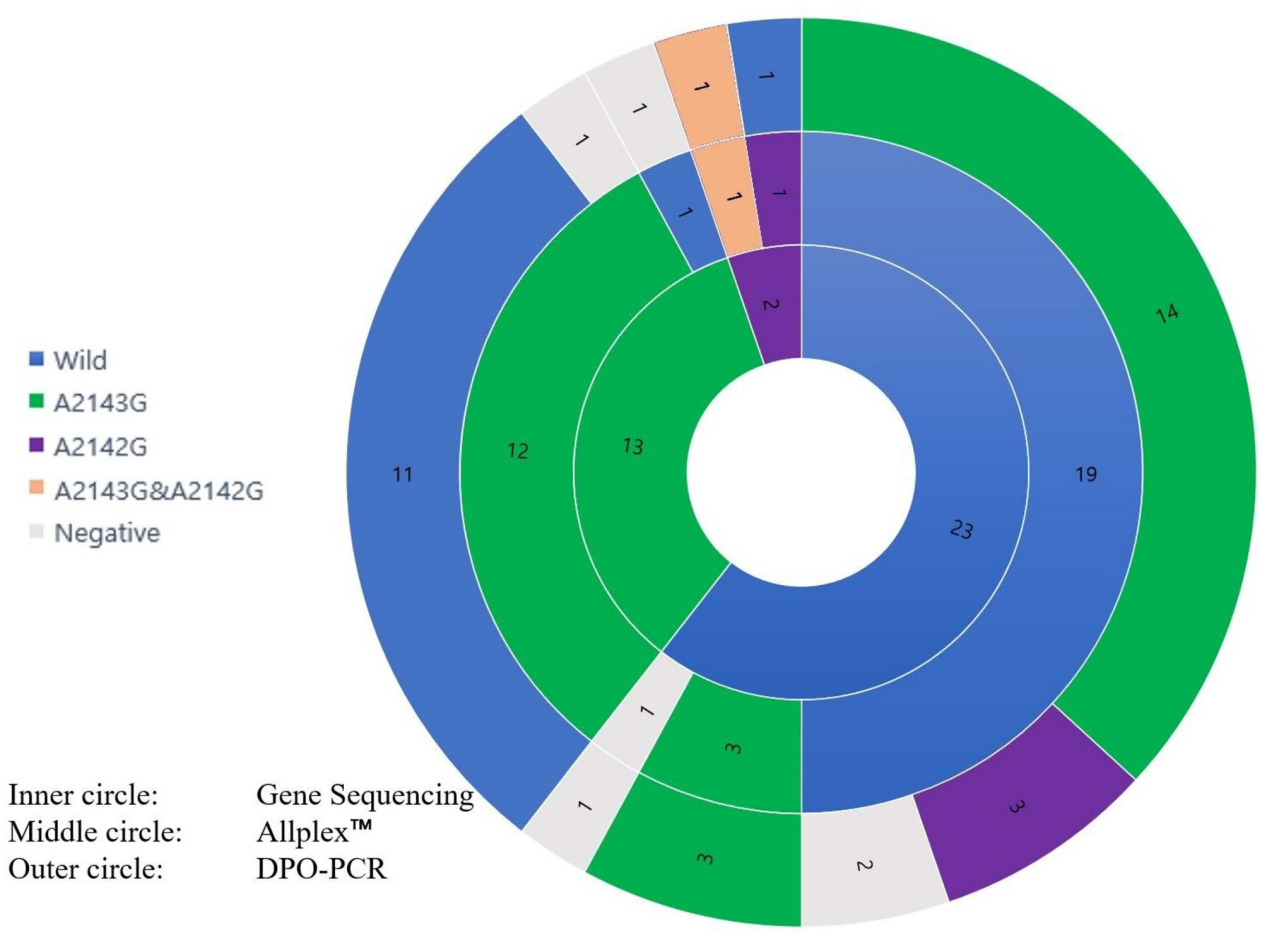
Summary of the discordant cases between gene sequencing and PCR tests. The frequency is described in each territory of tests and mutation types. Abbreviations: Allplex™, Allplex™*H pylori* and ClariR Assay; DPO-PCR, dual priming oligonucleotide-based multiplex polymerase chain reaction

DPO-PCR showed 104 concordances (58 wild types, 29 A2143G mutations, and 17 negatives) and Allplex™ showed 136 concordances (79 wild types, 41 A2143G mutations, 1 A2142G mutation, and 17 negatives) out of the 142 samples with gene sequencing. Among the 38 cases of disagreement by DPO-PCR, 12 cases confirmed as mutated by gene sequencing were confirmed as wild-type by DPO-PCR. However, Allplex™ showed only six cases of disagreement out of the 142 subjects, and only one case was mutated in gene sequencing among samples discriminated as wild-type by Allplex™. Allplex™ found 32 concordant test results with gene sequencing out of the 38 cases in which DPO-PCR didn’t agree with gene sequencing. (Fig. 2) On the other hand, DPO-PCR failed to detect any disagreement between the Allplex™ and gene sequencing.

## Discussion

Antibiotics susceptibility is the most important single factor for HP eradication, and therefore, the detection of resistance is essential. The gold standard for detecting antibiotic resistance is culture based. However, these methods require at least 20 ~ 72 h, low yield and limited accessibility due to their laboratory requirements. The culture methods are gradually being replaced by molecular biological methods such as PCR due to these limitations [14]. Recent PCR methods are faster, simpler, and more economical [15, 16]. PCR methods can find evidence of HP infection from the conventional stain or culture test-negative samples. The recent European guideline recommends clarithromycin susceptibility testing, if available through molecular techniques or culture, before prescribing any clarithromycin-containing therapy [5]. Guidelines recommend quadruple treatment when there is no information regarding clarithromycin resistance. The increased number of antibiotics could lead to disturbance of gut microbiota and resistance of other bacteria [17]. The PCR methods allow the use of the optimized triple therapy for 60–90% of patients. [5]

In South Korea, DPO-PCR is one of the available methods for testing clarithromycin resistance. Seeplex *H. pylori*-ClaR ACE Detection is a commercially available DPO-PCR kit that detect *H. pylori*, A2143G, and A2142G mutations. DPO-PCR showed its concordance with E-test regarding clarithromycin susceptibility in 95.3% of cases. [18] The eradication rates in clarithromycin-susceptible subjects determined by DPO-PCR were between 89.2 and 97.4%. However, for subjects with clarithromycin-resistant strains determined by DPO-PCR, Bismuth quadruple therapy is more effective with eradication rates of 91.7–93.5% for subjects with clarithromycin-resistance determined by DPO-PCR. [11, 19, 20]

Allplex™ is a recent multiplex quantitative real-time reverse transcription PCR (qRT-PCR) assay that uses multiple detection temperature techniques (MuDT) [21]. Compared to DPO-PCR, Allplex ™ is faster and likely to be more accurate because it does not need the western blot process. Allplex™ is commercially available in European Union countries, Brazil, Ukraine, and Kenya.

Among the 125 specimens with confirmed HP infection in gene sequencing, 99.2% and 96.0% were confirmed with Allplex™ and DPO-PCR, respectively. Allplex™ showed 100% sensitivity, 97.6% specificity, 98% PPV, and 100% NPV for detecting HP infection in a French study [12], similar to this study.

The point mutations by whole genome sequencing were well correlated with the clarithromycin resistance phenotype in a European study (congruence 99%)[22] In Korea, A2143G mutation revealed 85.7% of concordance with the phenotype (6 out of 7 strains)[10], and A2142G mutation showed 100% of concordance with the phenotype (20 out of 20 strains) [23]. These point mutations resulted in eradication failure with the clarithromycin-based regimen [23–25].

Compared to traditional diagnostic methods, both Allplex™ and gene sequencing exhibited a 0% rate of false negative, but DPO-PCR showed one false negative in detecting HP infection.(Table 5).

**Table 5:** Performance of Allplex™*H pylori* and ClariR Assay, DPO-PCR, gene sequencing and traditional diagnostic methods for detection of *Helicobacter pylori*

Giemsa or RUT	DPO-PCR	Allplex™	Gene Sequencing	Number of specimens
(+)	(+)	(+)	(+)	102
(+)	(-)	(+)	(+)	1
(-)	(+)	(+)	(+)	19
(-)	(-)	(+)	(+)	2
(-)	(-)	(-)	(+)	1
(-)	(-)	(-)	(-)	17

Abbreviations: Allplex™, Allplex™*H pylori* and ClariR Assay; DPO-PCR, dual priming oligonucleotide-based multiplex polymerase chain reaction; RUT, rapid urease test

For the detection of HP, both Allplex™ and DPO-PCR had a false positive rate of 0% and a false negative rate of 0.7% and 3.5%, respectively. For the detection of mutations, Allplex™ had a false positive rate of 2.8% and a false negative rate of 0.7%, while DPO-PCR had a higher false positive rate of 14.8% and a higher false negative rate of 5.6%. These results are compatible with those of other commercially available quantitative real time PCR assays [26, 27]. False results of PCR assays can be caused by a number of factors, including the electrophoresis process or the carry over DNA decomtamination process [28–30].

In this study, we compared the diagnostic performance of Allplex™ and DPO-PCR using gene sequencing as a reference test. Allplex™ showed better diagnostic performance for HP detection with a higher accuracy rate and kappa agreement levels. Allplex™ also showed better diagnostic performance for the detection of HP mutations. Our results suggest that Allplex™ can be considered for the diagnosis of HP and mutations.

This study has several limitations. First, the sample size was relatively small.

Second, this study did not investigate the phenotype of clarithromycin resistance with HP culture. However, many studies reported an almost 100% of match rate between genotype and phenotype of the point mutations A2142G, A2142C, and A2143G[22, 31] Third, A2142C mutation was not found in this study, confirming its low prevalence in Korea from a previous study, (1 out of 431 strains)[25] In the United states, the positivity rates of A2142G and A2143G mutations range from 5.3 to 14% and from 23.8 to 80%, respectively, and those of the A2142C mutation ranges from 0 to 4% [32–35]. In Europe, the A2142G and A2143G mutations range from 9.6 to 32.6% and from 25.0 to 44.1%, respectively, and A2142C mutation was reported to range from 1.6 to 1.9% [36, 37]. In Japan, the A2142G and A2143G mutations range from 2.0 to 2.3% and from 53.6 to 67.4%, respectively [38, 39]. Notably, most clarithromycin resistance cases in South Korea were from the A2143G mutation [40, 41].

Lastly, we did not study the other mutations which account for the clarithromycin resistance. Other than the three mutations in this study, A2115G, A2142T, G2141A, and T2182C were reported to influence clarithromycin resistance [42, 43]. However, in the previous studies, the most common mechanism of point mutations in clarithromycin resistance is preventing macrolides from binding sites were A2143G (69.8%), A2142G (11.7%), and A2142C (2.6%) [42].

In conclusion, Allplex™ showed comparable diagnostic performance with direct gene sequencing and non-inferior diagnostic performance to DPO-PCR. Further clinical research is required to confirm whether the increase in the diagnostic performance of Allplex™ leads to effective eradication therapy is effective.

## Data Availability

The data can be made available upon reasonable request from the Corresponding author.
