# Correction: Quantitative multiplex real-time polymerase chain reaction assay for the detection of *Helicobacter pylori* and clarithromycin resistance

**DOI:** 10.1186/s12866-023-02937-3

**Published:** 2023-07-15

**Authors:** Ilsoo Kim, Lee-So Maeng, Joon Sung Kim, Byung-Wook Kim, Dae Young Cheung, Jin Il Kim, Soo-heon Park

**Affiliations:** 1 Division of Gastroenterology, Department of Internal Medicine, Incheon St. Mary’s Hospital, College of Medicine, The Catholic University of Korea, Seoul, Republic of Korea; 2 Department of Hospital Pathology, Incheon St. Mary’s Hospital, College of Medicine, The Catholic University of Korea, Seoul, Korea; 3 Division of Gastroenterology, Department of Internal Medicine, Yeouido St. Mary’s Hospital, College of Medicine, The Catholic University of Korea, Seoul, Republic of Korea

## Correction

***BMC Microbiol*** **23, 155 (2023)**

10.1186/s12866-023-02868-z

Following publication of the original article [1], the authors noticed an inadvertent mistake regarding the affiliation of Dr. Ilsoo Kim, Joon Sung Kim, and Byeong-Wook Kim. The published affiliation reads as “Division of Gastroenterology, Department of Internal Medicine, Incheon St. Mary’s Hospital, College of Medicine, The Catholic University of Korea, Incheon, Republic of Korea”. However, the correct affiliation should be “Division of Gastroenterology, Department of Internal Medicine, Incheon St. Mary’s Hospital, College of Medicine, The Catholic University of Korea, Seoul, Republic of Korea”.

The mentioned affiliation in this erratum note has been updated accordingly.
